# Changes in Developmental Trajectories of Preschool Children with Autism Spectrum Disorder during Parental Based Intensive Intervention

**DOI:** 10.3390/brainsci10050289

**Published:** 2020-05-12

**Authors:** Arianna Bentenuto, Giulio Bertamini, Silvia Perzolli, Paola Venuti

**Affiliations:** Department of Psychology and Cognitive Science, Laboratory of Observation, Diagnosis and Educational (ODFLAB), University of Trento, 38068 Rovereto, Italy; giulio.bertamini@unitn.it (G.B.); silvia.perzolli@unitn.it (S.P.); paola.venuti@unitn.it (P.V.)

**Keywords:** Autism Spectrum Disorder (ASD), early intensive intervention, developmental trajectories, moderators and mediators of intervention.

## Abstract

Background: Research highlights the positive effects of early intensive intervention with parent and school involvement for preschool children with Autism Spectrum Disorder (ASD) on general developmental outcomes and social skills in randomized controlled trials. However, given the inter-individual variability in the response to treatment, it is necessary to investigate intervention effects in terms of mediators and moderators in order to explain variability and to highlight mechanisms of change. Methods: 25 children in the experimental group were exposed to early intensive intervention and 14 children in the control group were subjected to “as usual” intervention. The initial assessment was obtained at the time of diagnosis (T1) and the follow-up assessment was conducted after 15 months of intervention (T2) in both groups. Results: Participants in the experimental group achieved more prominent gains in both cognitive and socio-interactive skills. The role of specific factors able to predict general quotient and language quotient after intervention were investigated, pointing out the contribution of personal–social and performance abilities. Conclusions: The findings support the importance of parental involvement in targeting ASD core symptoms. Further, results informed our understanding of early predictors in order to identify specific elements to be targeted in the individualized intervention design.

## 1. Introduction

Autism Spectrum Disorder (ASD) is defined as a set of neurodevelopmental disorders (DSM-5) that impact on children’s development by disrupting socioemotional reciprocity and producing a set of restricted repetitive patterns of behaviours and interests [1]. According to the Centres for Disease Control, about 1 of 59 children were diagnosed with ASD [2]. Psychoeducational intervention for children with Autism Spectrum Disorder (ASD) currently represents a main strategy to achieve symptoms reduction, promoting better adaptation and developmental outcomes [3]. Therefore, the increased prevalence of ASD led to a growing attention to early intervention research. 

Different models of intervention started to prove their efficacy in randomised controlled clinical trials, together with longitudinally stable and generalizable outcomes [4,5,6,7,8]. Further, in line with this, a recent study review underlines how developmental interventions improve some specific areas, particularly socio-communicative domain in children with ASD [9]. Considering both efficacy and effectiveness of intervention, areas of improvement include IQ scores, verbal and non-verbal communication measures, adaptive behaviour and social and self-skills but there is less evidence of a significant impact on core autistic symptoms [10,11]. In line with this, specific improvement of core autistic symptoms has rarely been reported, mainly due to the lack of scalable and quantifiable autism-specific treatment response measures, and due to the fact that standardized diagnostic instruments are not sensitive enough to detect changes after intervention [12,13,14,15]. While overall group improvements may be evident, the rate and the nature of these improvements is highly variable across individual differences in children with ASD [16]. Studies on efficacy show, in fact, great inter-individual variability in the response. Some children respond well to treatment (high-responders), whereas other children respond less to the same model of intervention (low- or non- responders) [17,18]. Variability in ASD in fact, not only concerns clinical expressions but also intervention outcomes [19]. Hence, it is difficult to identify one kind of intervention with the highest degree of efficacy compared to others, given that a specific intervention can be useful for specific domains and patients but not for others [12,13,20]. Despite this, treatments share some common principles: precocity, intensity, individualisation and integrated work [20,21,22,23]. 

To conclude, a great amount of research reported the efficacy of different kinds of intervention, underlying improvement of specific skills and highlighting the fundamental role of personalisation. For this reason, current research is focused on developmental trajectories of children with ASD during intervention [24,25,26,27]. The role of specific factors influencing intervention response need further investigation [28]. Some evidence indicates that factors associated with different responses include pre-treatment cognitive abilities [10,19,29,30], symptoms severity [31], adaptive skills [30,32], younger age [33], communication abilities [34], play skills [35,36], interest in objects [37], joint attention [36] and imitation [31]. 

Overall, studies on developmental trajectories focused on cognitive and/or adaptive functioning and symptoms severity pointing out different trends. Cognitive and/or adaptive skills showed major improvements compared to symptoms severity that are demonstrated to be more persistent [19,38,39]. Further, there is consensus regarding the importance, as prognostic factors, of IQ and speech level measured at the beginning of intervention. The level of language development is an important variable that has long been considered a predictive factor of child’s outcomes [40,41].

In particular, children who received an intervention targeting early social intersubjective abilities have shown greater long-term language improvements than children in a control group [42]. Recent literature on developmental early intensive intervention focused mainly on interactive pleasure and exchange as a fertile ground to acquire competencies. In line with this, intervention intensity into the therapy room is not able to guarantee generalization of competencies if family and school are not encouraged to take an active role. Parents and school educators are, therefore, involved into the intervention program in order to generalize acquired competencies in more naturalistic settings. Further, there is some evidence that only children without intellectual disabilities at baseline were able to transfer the acquired socio-communication skills into daily life, therefore generalizing them [19]. In the Italian context, school represent a social opportunity in order to increase appropriate stimulations 

In order to investigate developmental trajectories, we considered the learning rates, calculated as the difference between mental ages before intervention and after intervention and the time elapsed. It represents an alternative tool to measure change in studies of early intervention [43]. Through these indexes, it is possible to compare developmental profiles throughout time, not only at an absolute level but also taking into account the time elapsed between the two assessments with regard to the typical developmental trajectory. It clearly represents changes in age-equivalents over time and it is more appropriate when intervention lengths of time are similar, but not perfectly equal. Further, it represents an advantage when children functioning’s are compared at different chronological ages. In fact positive learning rates mean that the child is narrowing the developmental gap. On the contrary, negative learning rates indicate a wider gap in the developmental trajectory. Learning rates may be useful for both outcome studies and progress representation of specific children functions [44], given that the value can be easily compared among them. 

For the reasons expressed above, the purposes of the present work were: (1) to compare developmental trajectories for children receiving a parental based intensive intervention that provides 5–6 h per week, with both family and school involvement, with children exposed to “as usual” intervention, that provides 2–3 h per week of rehabilitative activities delivered by community services (see Methods’ section for details); (2) to compare developmental trajectories of children with cognitive functioning equal or above 70 points at general quotient with children with cognitive functioning below 70 points at general quotient in both groups (3) to investigate the relationship between child pre-treatment characteristics and developmental trajectories. We had the following hypotheses in relation with the described objectives. First, we expected to find an overall increased level in cognitive abilities in both groups, however, we hypothesized a greater increase considering children exposed to early intensive intervention with family and school involvement, compared to children exposed to “as usual” intervention. Specifically, in relation to the intervention principles we hypothesized an increased level of linguistic skills. Secondly, we tried to identify a decreased level of autistic symptomatology, in particular considering the socio-communicative area, given the stability throughout the development of the restrictive and repetitive behavioural pattern [27,45]. Thirdly, consistently with previous studies [19,39], we expected that children without cognitive impairment showed major improvements in the developmental trajectory, compared to children with cognitive impairment. Finally, we hypothesized that specific child’s variables might influence the developmental trajectory, specifically the chronological age and linguistic abilities at the beginning of the intervention were considered. 

## 2. Materials and Methods

### 2.1. Participants

This study involved 25 children with Autism Spectrum Disorder (ASD) (M chronological age = 39.76 months, SD = 10.22; M mental age = 27.92 months, SD = 9.19) exposed to early intensive treatment with parent and school involvement delivered by ODFLab and 12 children with ASD (M chronological age = 45.33 months, SD = 8.34; M mental age = 33.17 months, SD = 12.80) subjected to “as usual” treatment delivered by community services in other regions after a diagnostic assessment at ODFLab (Table 1). All participants were recruited at ODFLab, a clinical and research centre of the Department of Psychology and Cognitive Science—(University of Trento) specialised in functional diagnosis of neuro developmental disorders, especially ASD, where families usually turn to in order to assess children’s clinical profile. Moreover, the laboratory employed and currently delivers early intensive intervention with a developmental perspective in the local community [46]. Families coming from other regions usually turn to ODFLab only for the first assessment and monitoring of developmental trajectories every year. The intervention is therefore carried out in their local community services. All families involved in this project were adequately informed about procedure and agreed with a written informed consent. They were also aware of the possibility to drop out from the study in every moment. 

The diagnosis of ASD was confirmed through clinical judgment by an independent clinician based on the DSM-5 criteria for Autism Spectrum Disorder, as well as through the Autism Diagnostic Observation Schedule (ADOS-2) [47]. 

The linguistic mental age was assessed through “Language and Communication subscale” of the Griffith Mental Development Scales. Considering the intervention group the average is 22.76 months (SD = 14.16) and for the control group the average is 27.75 months (SD = 13.51).

The socioeconomic status (SES) of the families, calculated with the Four-Factor Index of Social Status [48], indicated a middle status in the intervention group and a middle-high status in the control group. 

### 2.2. Procedure 

All procedures of our study were in accordance with the ethical standards of the Italian Association of Psychology (AIP) and with the ethical standards of the Ethics Committee of the University of Trento (Italy) and the last version of Declaration of Helsinki [49] 

In order to determine children’s developmental level, the Griffith Mental Development Scale-Edition Revised [50] was administered to all children. Children were classified as “children without cognitive impairment” if they received a score equal or above 70 on the general developmental quotient and as “children with cognitive impairment”, if they received a score lower than 70. In the experimental group, fourteen children (56%) were classified as children without cognitive impairment and 11 children (44%) were classified as children with cognitive impairment. Considering the control group, six children (50%) were classified as children without cognitive impairment and six children (50%) were classified as children with cognitive impairment. Taking into account the level of language development and the chronological age of children, ADOS Toddler, Module 1 and Module 2 were used to certify the presence of Autism Spectrum Disorder and to specify the severity level. 

These measures (see measures’ section for details) were applied before intervention (T1), during the first diagnostic and functional assessment. After intervention (T2), children were re-assessed in order to investigate developmental trajectories pre- and post-intervention, considering both cognitive and socio-interactive aspects. For participants in the experimental group (M = 14.72 months, SD = 4.36) and participants in the control group (M = 16.67 months, SD = 4.47) the amount of elapsed time, around fifteen months, is comparable.

### 2.3. Measures

#### 2.3.1. Griffiths Mental Development Scales-Edition Revised

The Griffiths Mental Development Scale, Edition Revised [50] was used to assess children’s mental development level. The GMDS-ER are developmental scales normalized also in an Italian sample and are administered by trained psychologists to the child in a laboratory setting through standardized activities designed to evaluate different aspects of mental development in infants and children, providing scores relative to 6 subscales: Locomotion; Personal–Social; Communication and Listening; Eye–Hand Coordination; Performance; and Practical Reasoning. This scale provides a global quotient and a developmental age-equivalent—allowing to detect developmental delays—as well as specific quotients and developmental age-equivalents for each of the 6 subscales. Both global score and subscale scores were taken into account for the purposes of the present study. 

#### 2.3.2. Autism Diagnostic Observation Schedule-2 (ADOS-2)

In the present study, we used the Autism Diagnostic Observation Schedule-2 (ADOS-2) [47] both to confirm participants’ diagnosis, to measure symptoms severity, and to investigate patterns of change before and after intervention. The administration of this tool is carried out by trained psychologists after an official ADOS course. For the purposes of this study, we used Toddler Module, Module 1 and Module 2. Each module gives a final score that classifies the child into mild, moderate or severe form of symptoms. Both global score and scores considering social-affect area and restricted, repetitive behaviours area are taken in consideration for the purposes of the present study.

### 2.4. Models of Intervention

#### 2.4.1. Parental Based Intensive Intervention

ODFLab (Observation, Diagnosis and Educational Laboratory) proposes and currently applies an “Italian Model of Intervention” which integrates empirically validated scientific principles together with guidelines in accordance to the Italian sanitary system and organization of educative system that guarantees a specialized educator for classrooms with children with special needs [22,46,51]. The intervention is individualized, comprehensive and integrates behavioural, developmental and relationship-based principles, according to the basic concepts of the Early Start Denver Model [10,13]. This intervention promotes Intentionality by giving to a child behaviour a communicative value so that he/she experiments that an action influences others behaviour and Reciprocity, starting from child behaviour to build up exchanges based on shift alternation. The therapist’s goal is, therefore, to facilitate intentionality and reciprocity for children and share them with parents and educators. Further, intervention goals are constantly monitored and changed depending on the child’s developmental improvements. Trained therapists aim constantly to create pleasant relationships starting from a child’s own pleasure during shared activities [22]. 

The intervention is focused on the activation of interactive circuits during communication and on acquisition of specific functional competencies through psycho-educative activities. The intervention identifies key target areas and comprises specific activities and related objectives that are progressively adapted based on a specific observational schedule. This is regularly filled in by the psychologists to monitor the learning trajectory and disclose emergent abilities to be targeted during the intervention. Hence, the activities are highly integrated into playful routines to promote the development of specific objectives (e.g., language) by means of a comprehensive work on emergent abilities (e.g., communicative gestures or imitation). These principles are in line with Early Start Denver Model and more generally with Naturalistic developmental behavioral interventions [9,10]. In order to strengthen the generalization of child competencies it is fundamental to involve caregivers into the therapeutic setting from the beginning. In fact, caregivers represent a child’s main interactive partners who, if they adequately learn appropriate interactive strategies, may effectively exploit them in more naturalistic settings. To this end, caregivers are involved in a child’s social routines as an active part during intervention. For the same reason, they are fundamental to help school educators in understanding and responding to child behavior and structuring adequate activities. Moreover, in the educational context, it is possible to implement peer-mediated routines to promote appropriate social exchanges with peers that usually are not included in rehabilitative and psycho- educative activities. The intervention comprises:

- for children: specific activities such as speech therapy, music therapy, cognitive activities and emotional and social play (4/6 h per week at the clinical centre)
- for parents: parent involvement into the therapy room (at least 2 h per week) and meetings every 15 days between therapist and parents through video feedback to provide adequate strategies to deal with children with ASD.
- for school: at the beginning, one hour per week with teacher and educator and the child in the school context. Then, meetings every three weeks with school educators in order to share specific interventions’ objectives and to organize play activities appropriately.

The focus of the proposed intervention is mainly on building the “net”; in fact, given the pervasiveness of the disorder, the treatment necessarily has to be multimodal, integrated, rooted in the community and it should provide the fundamental involvement of both family and subsequently of school. In order to promote generalization of child’s competencies, the network is aimed at providing appropriate strategies to detect and promptly respond to the child’s needs, decreasing the child’s frustration and boredom. 

The intervention is delivered by licensed psychologists after receiving specific training on developmental models of intervention for children with ASD. The team is regularly supervised at least once every three weeks by an expert psychotherapist and all the psychologists have completed the introductory course to the Early Start Denver Model. Further, some of them attended the advanced course. 

#### 2.4.2. “As Usual” Intervention 

With the term “as usual” intervention, we refer to specific rehabilitative activities such as psychomotricity and speech therapy employed by local community services. In particular, psychomotricity comprises a set of activities to promote communicative and relational abilities by means of body awareness and body movement. Psychomotricity is performed by professionals with a specific bachelor’s degree. Moreover, speech therapy directly targets receptive and expressive language without a specific focus on socio-communicative routines. These specific activities represent effective strategies for intervention with preschool children with ASD [46] The intensity is generally from one to three hours per week, calibrated according to child’s needs by the reference developmental neuropsychiatrist [46]. In the community services, no active involvement of caregivers and school is provided, but meetings for parents are planned if requested by them and two institutional meetings per year are planned with school educators to monitor the child’s schooling. 

From the two interventions’ description, we would like to underline that the core difference regards the degree of involvement of social context families and school and not the specific rehabilitative activities known to be effective in dealing with children with ASD. 

## 3. Results

### 3.1. Analytic Plan

The data were controlled for normality and homoscedasticity through the Shapiro–Wilk normality test and Levene test for homogeneity of variances. Parametric inferential tests (T test) were used when appropriate to identify group differences before the intervention (T1) and after the intervention (T2), as well as for investigating longitudinal changes. Otherwise, non-parametric tests were performed (Wilcoxon–Mann–Whitney test). Effect sizes were calculated using r^2^. Linear Regression models were implemented to test for predictors of change, and checked for assumptions. Repeated Measures Analyses of Variance (ANOVA) were performed to check for Group differences. Data were analysed using R statistical software [52].

### 3.2. Preliminary Analysis

At T1, there were no significant differences in chronological ages between the intervention group (M = 39.76 months; SD = 10.22) and the control group (M = 45.33 months; SD = 8.84), and the time passed between the first and the second assessment was not significantly different between the two groups (t(35) = 1.26 ; *p* = 0.215; r^2^ = 0.044). Further, no significant differences (t(31) = 1.630; *p* = 0.113; r^2^ = 0.08) emerged between the intervention and the control group regarding the socio-economic status of the families.

There were no significant differences at T1 and T2 between the two groups also regarding age equivalents of all the subscales of the Griffiths Mental Development Scales, as well as standardized quotients and the Autism Diagnostic Observation Schedule-Second Edition scores (Table 2 and Table 3). Therefore, the whole sample was included to fit linear models. Then, paired T tests in both groups were performed to identify longitudinal changes.

### 3.3. Longitudinal Changes

#### 3.3.1. Cognitive Profile 

Paired T-tests for dependent samples revealed a significant (t(24) = −2.320; *p* = 0.029; r^2^ = 0.18) change in the General Quotient of the Griffiths Mental Development Scales between T1 (M = 73.64; SD = 15.84) and T2 (M = 79.12; SD = 22.02) for the intervention group. Children in the intervention group had a mean difference of 5.48 (SD = 11.81). The control group showed a non-significant (t(11) = −1.52; *p* = 0.156; r^2^ = 0.17) longitudinal change between T1 (M = 74.08; SD = 19.5) and T2 (M = 69.50; SD = 18.28) in the General Quotient, with a mean difference of 4.58 (10.43).

Regarding the longitudinal changes for Locomotor, Personal-Social, Performance and Practical Reasoning subscales, no significant differences emerged between the intervention and control groups. However, the control group showed a significant (t(11) = −2.434; *p* = 0.033; r^2^ = 0.350) improvement in the Eye and Hand Coordination subscale between T1 (M = 64.00; SD = 17.73) and T2 (M = 73.25; SD = 17.32). The change between T1 (M = 72.80; SD = 18.87) and T2 (M = 78.12; SD = 22.43) resulted to be non-significant (t(24) = −1.77; *p* = 0.089; r^2^ = 0.115) in the intervention group.

The Language Quotient showed a significant (t(24) = −3.387; *p* = 0.002; r^2^ = 0.32) change between T1 (M = 58.00; SD = 28.97) and T2 (M = 75.32; SD = 35.34) in the intervention group with an effect size indicating a strong effect in this subscale. Children in the intervention group had a mean difference of 17.32 (SD = 25.57), showing strong improvements in the Language domain. The difference was significant (t(11) = −2.59; *p* = 0.02; r^2^ = 0.38) also for the control group, showing a mean difference of 9.58 (SD = 12.82), lower than the intervention group. (Table 2) 

#### 3.3.2. Socio-Communicative Profile

A significant (t(24) = 4.50; *p* = 0.0001; r^2^ = 0.46) difference in the ADOS-2 Total Score emerged between T1 (M = 16.20; SD = 4.15) and T2 (M = 13.60; SD = 4.33) in the intervention group, indicating a strong effect size. The difference was resulted to be non-significant (t(11) = 1.73; *p* = 0.112 r^2^ = 0.21) in the control group, with a mean difference of -1.58 (SD = 3.18) and a lower effect size. Regarding the intervention group, a significant (t(24) = 4.08; *p* < 0.001; r^2^ = 0.41) difference in the Social Affect area between T1 (M = 12.32; DS = 3.18) and T2 (M = 10.04; DS = 3.35) emerged, indicating a strong effect and a mean reduction of -2.28 (SD = 2.79). A significant (t(11) = 2.80; *p* = 0.017; r^2^ = 0.42) difference between T1 (M = 11.75; SD = 3.55) and T2 (M = 10.08; SD = 3.48) was also found in the control group, with a mean difference of −1.67 (SD = 2.06). (Table 3)

### 3.4. Children with and without Intellectual Impairment

Afterwards, to further investigate trajectories of change, the sample was differentiated in terms of cognitive functioning between the two groups. Coherently with literature and clinical standards, the threshold of 70 was considered in the General Development Quotient of the Griffiths Mental Development Scales. The filter yielded 14 children with General Quotient above 70 in the intervention group (11 children with General Quotient equal to or below 70) and six children above 70 in the control group (six children equal to or below 70). 

Regarding the General Quotient, the children without intellectual impairment in the intervention group showed a significant (t(13) = −3.71; *p* = 0.003; r^2^ = 0.51) longitudinal difference between T1 (M = 84.64; SD = 10.87) and T2 (M = 96.14; SD = 8.69) indicating a strong effect with a mean difference of 11.5 (SD = 11.61). This difference was resulted to be non-significant (t(5) = −1.41; *p* = 0.219; r^2^ = 0.28) in the control group between T1 (M = 82.83; SD = 6.77) and T2 (M = 87.67; SD = 9.77), with a mean difference of 4.83 (SD = 8.42) and a lower effect size.

Focusing on the Language subscale, children in the intervention group with a General Quotient above 70 at T1 showed a significant (t(13) = −4.00; *p* = 0.002; r^2^ = 0.55) longitudinal difference between T1 (M = 73.79; SD = 29.54) and T2 (M = 102.14; SD = 17.84), indicating a strong effect with a mean difference of 28.36 (SD = 26.53). Children in the control group who had a General Quotient above 70 showed a non-significant (t(5) = −1.97; *p* = 0.106; r^2^ = 0.44) difference between T1 and T2 in the Language Quotient. The effect size was still relevant, but the mean difference was 11.67 (SD = 14.50). The difference was resulted to be non-significant between the two groups with respect to children with intellectual impairment.

With respect to the ADOS-2, a significant (t(13) = 4.09; *p* = 0.001; r^2^ = 0.56) longitudinal difference emerged in the Total Score in the intervention group without intellectual impairment between T1 (M = 11.29; SD = 3.00) and T2 (M = 8.14; SD = 2.60), indicating a strong effect with a mean difference of −3.14 (SD = 2.88). The difference was not significant in the control group of children without intellectual disability (t(5) = 1.6; *p* = 0.17; r^2^ = 0.34) with a mean difference of −2.67 (SD = 4.08) and a lower effect size. 

No significant differences emerged with respect to the Repetitive Restricted Behaviors area in both children with and without intellectual disability.

Furthermore, considering the Social Affect area, a significant longitudinal difference (t(13) = 3.69; *p* = 0.003; r^2^ = 0.51) emerged for children without intellectual impairment in the intervention group between T1 (M = 11.29; SD = 3.00) and T2 (M = 8.14; SD = 2.60), indicating a strong effect and a mean difference of -3.14 (SD = 3.18). The difference was not significant for children without intellectual impairment in the control group (t(5) = 2.15; *p* = 0.08; r^2^ = 0.48), with a mean difference of −2.33 (SD = 2.66). With respect to children with intellectual impairment, no significant differences emerged between the two groups.

### 3.5. Predictor Analysis

In the analysis of predictors of outcomes, all participants were considered without group distinction, given that all children received some form of intervention. Linear Regression Models were fitted in order to test the goodness of different sub quotients at T1 in predicting the General Quotient and the Language Quotient at T2.

The General quotient at T2 was predicted by the combination of Personal-Social (β = 0.46; *p* = 0.006) and Performance Quotients (β = 0.21; *p* = 0.041) and the Chronological age (β = −0.60; *p* = 0.003) at T1. The model was significant (F(4,32) = 27.38; *p* < 0.001; Adjusted R^2^ = 0.75) and explained a significant proportion of the variance. The Language Quotient term resulted to be not significant (β = 0.18; *p* = 0.082) in this model.

Then, the Language Quotient at T2 was considered as a dependent variable and possible predictors among the subquotients at T1 were investigated. The Language quotient at T2 was predicted by the Language Quotient (β = 0.67; *p* < 0.001), the Personal-Social Quotient (β = 0.50; *p* = 0.036) and the Chronological age (β = −1.12; *p* = 0.001) at T1. The model resulted to be significant (F(3,33) = 28.74; *p* < 0.001; Adjusted R^2^ = 0.70) and explained a significant proportion of the variance.

### 3.6. Responders and Non-Responders

The 41% of the total sample responded to the interventions with a recovery in the age-equivalent, having a positive learning rate. This group was defined as “responders”. In particular, in the intervention group, there was a percentage of 44% of responders, while the control group had a 25% of responders.

To investigate the baseline characteristics of children who positively responded to the intervention, differences at T1 between the responders and non-responders groups were examined.

The General Quotient of the responders group (M = 79.36; SD = 8.85), was significantly (t(35) = −2.12; *p* < 0.05; r^2^ = 0.11) higher than the General Quotient of the non-responders group (M = 68.00; SD = 18.70) at T1. 

Considering the sub quotients, only the Language Quotient of the responders group (M = 69.86; SD = 24.27) was significantly (W = 80; *p* = 0.012) higher than the Language Quotient of the non-responders group (M = 52.00; SD = 27.71) at T1.

Considering the age-equivalents learning rate in the Personal-Social domain, a significant (t(35) = 3.90; *p* < 0.001; r^2^ = 0.30) difference emerged at T1 in the ADOS-2 score of Repetitive Restricted Behaviors between the responders and non-responders groups. Responders group started with a mean score of 2.31 (SD = 1.49), while the non-responders group had a mean score of 4.54 (SD = 1.74).

Moreover, a significant (t(35) = −2.25; *p* < 0.05; r^2^ = 0.13) difference emerged at T1 in the Personal-Social Quotient between children who showed improvements in the age-equivalents learning rate of the Language subscale. The responders group started with a mean Personal–Social Quotient of 76.94 (SD = 16.81), while the non-responders group had a mean of 62.14 (SD = 21.80) at T1.

Finally, considering the age-equivalents learning rate in the Performance subscale, children who improved in time (responders) started with a mean Personal-Social Quotient of 77.19 (SD = 16.98), whereas non-responders group had a mean quotient of 61.95 (SD = 21.56) at T1. The difference was significant (t(35) = −2.33; *p* < 0.05; r^2^ = 0.13).

## 4. Discussion

Given the complexity of evaluating treatment efficacy and the importance of individualized treatment for children with ASD, the main purpose of the present study was to analyse developmental trajectories of preschool children with ASD in order to understand how specific developmental areas evolve in time. As a way to do so, we took into consideration two groups of children exposed to two different kinds of intervention. On the one hand, an intensive intervention focused on the involvement of family with a specific work on wide-range socio communicative abilities and on the other hand, a rehabilitative “as usual” intervention. The results of the empirical research underline how early intensive intervention with parent involvement promotes better results and generalization of a child’s competencies [4,5].

Regarding our first aim concerning the differences in the trajectories, our results are in line with the previous literature [5,6,10,22]. In fact, a significant improvement in the general quotient of children exposed to the early intensive intervention emerged, compared to children receiving the rehabilitative “as usual” intervention. 

In particular, analysing the specific subscales, it came to light that linguistic-communication abilities present major improvements compared to the other subscales of the general quotient for both groups. In fact, the significantly increased level in the control group is not surprising given that specific rehabilitative activities provided also by local community services improve child linguistic abilities, especially considering both receptive and expressive language. In line with this, the recent literature, using a different measure for investigating the general quotient (Mullen Scales of Early Learning, Communication and Behavior Scales), reported major improvements in linguistic and communicative areas, particularly in both expressive and receptive language after 9 months [45]. 

Further, our results support the ground idea of developmental models of treatment for ASD that, unlike specific rehabilitative speech therapy-centred treatment, focus on wide-range socio communicative abilities. Developmental models of intervention [4,43] are based on the exploitation of communicative nonverbal behaviours, gestures and their integration together with intentionality and reciprocity to promote the development of language skills through generalization and reduction of avoidance of social interactions. Interestingly, in our intervention group, the mean difference in language skills between the two assessments was greater than the mean difference of the control group. One possible explanation of this result derives from theoretical principles of the intervention that focus on developmental phases with the major aim of promoting intersubjectivity during the exchanges with the other (e.g., supporting non-verbal communication and the correct understanding of social signals). To this end, intentionality and reciprocity are promoted given their importance for language development [40,41]. Further, these results could also be explained by the specific features of the intervention proposed. In fact, the intervention design is aimed to impact the most possible different contexts in the daily living of the child, and greatly extends the possibilities to experiment effective social interplays in a wider range of contexts. In our idea, participating at a major numbers of more appropriate social interactions could lead to better outcomes for children.

From the analysis of the cognitive profile, a significant increase in eye-hand motor coordination for the “as usual” intervention group also emmerged. In fact, a possible explanation could be that rehabilitative interventions such as psychomotricity comprise focused and specific motor activities, involving both gross and fine motor skills. From a clinical point of view of integrating different modalities in order to reach major outcomes, it is important also for networking interventions to comprehend rehabilitative activities to support these aspects. 

Concerning the socio-communicative area, that is our second hypothesis, our analysis shows that the general behavioural expressions of ASD decreased significantly in both groups. In fact, some atypical behaviours tended to diminish after the intervention. In particular, children showed improved competencies in the socio-communicative area [9]. These gains were more prominent in the early intensive intervention group, probably thanks to active involvement in the social context that guarantees a generalization of competences. Furthermore, in line with the literature, the area of restrictive and repetitive behaviours tends to be more stable. In fact, previous studies did not find significant modifications regarding this area after intervention [45,53]. Interventions generally support specific cognitive and social abilities that do not directly impact the area. Furthermore, the specific trends of this domain appear to be under-investigated [54]. However, slight modifications in this area, like the reduction emerged in the intervention group in our results, could be related to the specific work on anxiety reduction, emotions and self-regulatory mechanisms.

Taken together, these results highlight how specific work on a wide range of socio communicative abilities could promote better linguistic gains together with a reduction in symptoms severity with respect to the Social Affect area of the ADOS-2. Interestingly, this area of the ADOS-2 focuses on communicative abilities and social affect, considering different modalities and their integration. These results support the idea that intervention impacts developmental trajectories improving a large spectrum of socio communicative abilities, including receptive and expressive communication but also those important precursors of verbal communication like gestures, imitation and joint attention, fundamental elements to initiate or respond adaptively to the social exchange.

There is great consensus regarding the importance of cognitive level as a prognostic factor considering the developmental trajectory of children with ASD. References [38,39] also pointed out that children with cognitive level equal or above 70 points at the general quotient tend to improve more rapidly over time. In line with this, cognitive abilities are associated with different outcomes. For example, [19] found out that only children without impairment gained significant improvement in adaptive skills after 2 years of treatment, compared to children with intellectual disability. Further, only the first group of children was able to transfer the acquired socio-communication abilities into daily life after 1 year of treatment, showing generalization of competencies. On the contrary, this was not found for children with intellectual impairment. In line with these findings, our results show that children without intellectual impairment in the intervention group reached major gains in the general quotient after intervention. Particularly, the same pattern emerged considering the linguistic quotient, in which children without intellectual disability in the intervention group showed major improvements compared to the other group. With regard to children with cognitive impairment, no differences in both early intensive intervention and “as usual” groups were found.

Another key aspect focusing on developmental trajectories of symptom severity revealed that children without intellectual impairment show a more relevant increase in socio-communicative competencies compared to children with intellectual impairment [38,39]. In line with the analysis considering symptomatology of ASD, our results point out two different trajectories in the group exposed to early intensive intervention with parent involvement: less variability in symptoms expression was found considering children with cognitive impairment, and more gains were found regarding children without intellectual disability. With respect to the group exposed to the early intensive intervention, we found a specific trajectory that characterized children without intellectual impairment: increased level of cognitive abilities, specifically concerning linguistic skills, and reduced levels of symptoms expression. This specific outcome profile was coherent with one specific trajectory defined by [38].

A debate is still open on the identification of pre-treatment variables associated with different response outcomes.

With respect to our third aim, chronological age at the beginning of the intervention had an important role in predicting developmental outcomes, strongly supporting the idea of early intervention with children with ASD. Further, the analysis of pre-treatment variables pointed out the personal–social and performance areas as important predictors of the general quotient after intervention. In our analysis, younger children with better nonverbal intelligence skills, assessed by the Performance subscale, and personal autonomies (assessed by the Personal-Social subscale), showed better developmental outcomes. To our knowledge, no previous studies investigated the relationship between different domains of development and subsequent outcomes. Interestingly, our results highlighted the association of two specific developmental areas as possible prognostic markers of better developmental trajectories. 

A wide consensus is present concerning chronological age, supporting early intervention [6,23]. However, the relation with cognitive functioning appears to be more complex, with controversial evidence. On the one hand, lower cognitive skills are found to be associated with larger improvements [55], pointing out the possibility of substantial improvements for children starting below the average. On the other hand, other authors found out that higher cognitive skills predicted better outcomes on child developmental trajectory [39,56], suggesting a complex relationship that needed to be further investigated. More interestingly, sub-components of the general intelligence were investigated to identify markers in the neurodevelopmental profile and early neurodevelopmental milestones that could predict later cognitive functioning and the acquisition of language [40].

With the aim to deeply analyze developmental domains and given the significant improvement concerning language skills in our results, we focused the analysis on the Language Quotient after intervention, showing that pre-treatment language skills and personal-social abilities, together with age, predict better linguistic outcomes. This could underlie how, in the development of language, an important role is played by nonverbal communicative aspects [57]. In fact, the Personal–Social subscale investigates the development of a wide range of nonverbal communicative and social signals (e.g., social smile, showing, orienting the others’ gaze and communicative gestures) whereas in the Language subscale, besides the verbal skills, another set of communicative behaviours (e.g., pointing) are investigated, supporting the idea that the association between these two factors could represent possible prognostic markers specific for language development. 

Taken together, and in line with other recent research works [40], these results seem to support the impact of wide-range of socio communicative behaviours and skills on developmental trajectories, regarding both the general cognitive skills and, more specifically, on language development [58]. Further, despite previous research depicting the role of symptom severity on intervention outcome, our analysis suggests that developmental areas were more predictive of outcomes than symptom severity before the intervention [26,40].

On the basis of these results, the analysis of responders focused on differentiating children who recovered in age-equivalents, narrowing the gap between their chronological and mental age, from children who seem to remain more stable. Interestingly, the responders showed a higher cognitive functioning before intervention and, in particular, greater language skills, coherently with our previous results. Furthermore, children who narrow the linguistic gap started with higher personal–social abilities and, interestingly, children who closed the performance gap also started with higher personal–social abilities. These results highlight the role of some cognitive factors (in particular, personal–social skills) not only in predicting outcomes after intervention but also in differentiating children who showed significant recovery from those characterized by more stable response trajectories. 

Finally, concerning the trajectory of symptoms severity, our results evidence a significantly higher proportion of children who showed a reduction in symptomatology in the intervention group. Unexpectedly, a significant difference in restricted and repetitive behavioural pattern before intervention emerged between children who show a better recovery in personal–social skills, being characterized by lower symptom severity, and children who show a more stable outcome in this cognitive domain. This result may point out a potential role of this area in supporting or impeding the development of personal–social abilities and require further investigation in order to better understand its impact on the developmental trajectory. 

This knowledge may have important implications for clinical practice, providing clinicians more information about specific areas to be targeted by the intervention and disclosing the importance of specific behaviours for subsequent language outcomes.

### Limitations 

This study presents some limitations. First of all, despite our results being in line with previous literature, a main limitation of the present work is represented by the small sample size, and hence, results should be replicated in studies with larger samples. Further, sample size is important with respect to the high variability reported in the literature concerning different response trajectories. A small sample size reduces the possibility to investigate clusters of response profiles [39]. Moreover, the sample is unbalanced with respect to gender, thus reducing the possibility to investigate gender differences in the response trajectory, as emerged by recent literature [59]. In addition, our sample was not randomized. However, our aim was to understand intervention outcomes guarantying to patient better opportunities with respect to the specific intervention offered by the local territories. Children were assessed by independent examiners that were aware of their local origin but blinded to this study and not involved in children’s therapeutic intercourse. The presence of only two assessments represents a limitation in order to better evaluate the response trajectory. Thus, an additional point to address in our further studies will be to measure children’s developmental profiles in other time points in order to trace the response during time evidencing improvements and tendencies towards the stabilization of the profiles. Another future perspective is represented by a detailed analysis of specific socio-communicative elements evaluated by the ADOS-2. As an example, social affect behaviours such as pointing, showing and quality of social overtures could be important markers of change to be investigated, as pointed out by some research results [58], and could play a role in the response. Finally, characterizing children who narrow the gap and those displaying more stable trajectories could better inform about prognostic markers associated with better outcomes. In addition, it could disclose new features to be taken into account in order to explain the variability in the response and improve developmental outcomes of more persistent profiles.

## 5. Conclusions

Identifying early trajectories of children with ASD has both theoretical and clinical implications. From a theoretical perspective, it can inform our understanding of early predictors and mediators of change in order to identify specific elements to be targeted in the intervention design. Further, this type of perspective enhances knowledge about ASD according to a developmental perspective. 

From a clinical standpoint, careful attention to developmental trajectories may help in structuring individualized intervention based on a child’s specific competencies in every phase of development. Finally, it is important to emphasize the fundamental role of social context in order to guarantee generalization of child competencies and better outcomes over time.

To conclude, the importance of networking intervention on child cognitive and social development led us to exploit online technologies in order to support social context through regular meetings to build up a valid online network. 

## Figures and Tables

**Table 1:** Demographic statistics.

	Intervention Group M (SD)	Control Group M (SD)
Chronological age (months)	39.76 (10.22) range (23–46)	45.33 (8.34) range (34–59)
Mental age (months)	27.92 (9.19) range (14–56)	33.17 (12.80) range (9–54)
SES (index)	36.36 (13.90)	46.69 (20.35)

**Table 2:** Developmental quotients in the two groups at T1 and T2.

	Intervention Group (T1) M (SD)	Intervention Group (T2) M (SD)	INT T1 vs. INT T2	Control Group (T1) M (SD)	Control Group (T2) M (SD)	CNT T1 vs. CNT T2
General Quotient	73.64 (15.84)	79.12 (22.02)	t(24) = −2.320 *p* = 0.029 * r^2^ = 0.18	69.50 (18.28)	74.08 (19.51)	t(11) = −1.52 *p* = 0.156 r^2^ = 0.17
Locomotor Quotient	79.08 (18.54)	79.68 (19.85)	t(24) = −0.234 *p* = 0.817 r^2^ = 0.002	83.50 (21.33)	76.75 (15.05)	t(11) = 1.924 *p* = 0.081 r^2^ = 0.25
Personal-Social Quotient	70.36 (21.79)	75.04 (21.27)	t(24) = −1.52 *p* = 0.142 r^2^ = 0.088	64.75 (19.27)	71.33 (17.19)	t(11) = −1.555 *p* = 0.148 r^2^ = 0.180
Language Quotient	58.00 (28.97)	75.32 (35.34)	t(24) = −3.387 *p* = 0.002 ** r^2^ = 0.32	60.33 (25.49)	69.92 (29.70)	t(11) = −2.59 *p* = 0.02 * r^2^ = 0.38
Eye-Hand Coordination Quotient	72.80 (18.87)	78.12 (22.43)	t(24) = −1.77 *p* = 0.089 r^2^ = 0.115	64.00 (17.73)	73.25 (17.32)	t(11) = −2.434 *p* = 0.033 * r^2^ = 0.350
Performance Quotient	86.76 (23.38)	89.40 (24.23)	t(24) = −0.690 *p* = 0.497 r^2^ = 0.019	81.33 (27.89)	85.50 (23.96)	t(11) = −0.791 *p* = 0.446 r^2^ = 0.054

* *p* < 0.05; ** *p* < 0.01.

**Table 3:** ADOS scores in the two groups at T1 and T2.

	Intervention Group (T1) M (SD)	Intervention Group (T2) M (SD)	INT T1 vs. INT T2	Control Group (T1) M (SD)	Control group (T2) M (SD)	CNT T1 vs. CNT T2
Social Affect Score	12.32 (3.18)	10.04 (3.35)	t(24) = 4.08 *p*<0.001 ** r^2^ = 0.41	11.75 (3.55)	10.08 (3.48)	t(11) = 2.80 *p* = 0.017 * r^2^ = 0.42
Restricted Repetitive Behaviors	3.88 (1.64)	3.56 (1.76)	t(24) = 0.902 *p* = 0.376 r^2^ = 0.033	3.50 (2.58)	3.75 (1.76)	t(11) = -0.353 *p* = 0.731 r^2^ = 0.011
Total ADOS-2 Score	16.20 (4.15)	13.60 (4.33)	t(24) = 4.40 *p* = 0.0001 *** r^2^ = 0.46	15.42 (5.09)	13.83 (4.73)	t(11) = 1.73 *p* = 0.112 r^2^ = 0.21
Severity Index	6.40 (1.63)	5.84 (1.37)	t(24) = 1.937 *p* = 0.065 r^2^ = 0.135	5.92 (1.78)	5.42 (1.78)	t(11) = 1.483 *p* = 0.166 r^2^ = 0.167

* *p* < 0.05; ** *p* < 0.01; *** *p* < 0.001.
